# Verified Provings of Oxygen

**Published:** 1890-09

**Authors:** S. Swan

**Affiliations:** New York


### VERIFIED PROVINGS OF OXYGEN.

S. Swan, M. D., New York.

*First proving.*—Dr. Swan took *Oxygen*$^{200}$ (Fincke); date and repetition of dose omitted. Since then has been notably troubled with great flatulence, passing at stool large quantities; the flatus seems to accumulate in rectum; does not generally notice it except from its producing a desire for stool; fears to pass it when not at stool, from an impression that stool will pass.

Great quantity of indurated mucus in nose in all stages of hardness, causing frequent necessity to pick it, and in morning, blowing out of lumps which are generally tough, opaque, whitish-yellow.

*Second proving.*—March 17th, took one dose of 1M (Fincke). Canker sores in mouth and cheeks.

March 19th.—Slight hoarseness and dryness of throat; toward evening the dryness increased round rim of glottis. Took *Acid-lac.*$^{1m}$ without benefit; becoming more annoying, took *Bell.* with partial relief. At midnight woke with choking, burning dryness of glottis and upper larynx. No thirst.

March 20th.—Early in morning took Acid-lac.$^{10m}$ and the dryness was entirely relieved, but was followed by great hoarseness and a hard, shaking cough, with considerable expectoration, excited during night by tickling under sternum, upper half, aggravated while lying on either side, relieved while lying on back; the cough was shaking, tearing, with profuse, lumpy, tasteless, whitish expectoration. Sweat all over, and headache between eyes.

March 21st.—Headache in right eyebrow, outer half; pain still in frontal region. Expectoration occasionally—yellow, purulent. Continued perspiration on scalp; loss of smell in a great degree. Aphonia, and difficulty in controlling the voice. Pulse ninety-two.

March 22d.—With dry, hacking cough last night, took *Rumex*$^{1m}$ and slept till two A. M. Cough then commenced from the same tickling, low down in supra-sternal fossa, and abundant expectorations, mostly of thick, white, tasteless, hard lumps; the cough caused great soreness in the muscles of the epigastrium. After two hours it ceased, with the exception of an occasional cough. There was perspiration on the scalp and slight moisture over the body.

March 23d.—Woke again at two A. M. and coughed till five A. M.; the same expectoration, white thick-like curds or yellow, creamy, tasteless, mixed with blood; this, however, probably came from the teeth, as they are in the habit of bleeding. There is not so much soreness in the region of the exterior attachment of the diaphragm, perhaps owing to a dose of *Kali-bichrom.* which I took before retiring. Dull headache over frontal region, more intense in a spot over left eye. Pain in left temple, which feels cold to the touch. Occasional cough during the day, with expectoration of white mucus if the cough is not severe, but of thick, white curds or yellow, creamy mucus when the paroxysm is more profound. Continual tickling in supra-sternal fossa. Took *Kali-bichr.*$^{1m}$ to-night.

March 24th.—Slept better, and did not cough to wake me till seven A. M.—coughed and raised a large amount of mucus. During the day single coughs not very often, always with expectoration, sometimes spasmodic, forcing the mucus into posterior nares, and causing hawking and blowing the nose. Having been exposed during evening to sleet and east wind, I coughed considerably on retiring, the cough being particularly distressing, causing a sensation as if the chest would burst at the lower part, with a tearing, sore sensation. It seems as if the interior attachment of the diaphragm would give way. There is a continuous dryness in throat, causing inclination to cough, frequent sneezing, and constant frontal headache, sometimes pressing downward over root of nose.

March 25th.—Rose very unwell from sore feeling in chest and dryness of throat and headache. Sneezes and raises large quantities of transparent, slimy, tasteless mucus; later in morning the expectoration was like white curds. Pain in ball of right eye and little to left of pupil—lancinating, paroxysmal. Occasional rush of pain, filling the whole ball of the right eye and extending to right temple, which then became hot.

March 26th.—Coughed on lying down last night, but did not raise much; in a short time fell asleep and slept till morning. Coughed considerably, not so much from irritation, but from the large quantity of mucus, mostly white and slimy. Some sneezing. Occasional pains in right eye and temple.

April 10th.—The cough, with the above symptoms, continued till now. Expectoration principally in the morning. Cough on lying down at night, caused by the sensation of a clot of mucus in the trachea or near the bifurcation of the bronchia, which, when loosened and raised, relieves the cough.

Eruption of fine stinging rash on right side of scrotum, continuing one day. Eruption of pimples in the fold of the nates, right side near anus becoming very sore, and seemingly like blisters, as the skin rubbed off, leaving it very sore. Takes cold easily; cough aggravated by exposure to the wind whether dry or damp.

April 13th.—Took one dose of 1 M.

During April cough gradually decreased. Desire for stool resulted only in flatus or small, bad-smelling stool. Itching of the skin on the metacarpal end of first phalanx of left index finger, no redness or eruption.

Painful stricture or tightness under centre of sternum, aggravated by bringing the shoulders forward.

### CLINICAL VERIFICATIONS.

(1). A. S., aged twenty-one and a half, severe cough from dryness in upper larynx and constant tickling in throat-pit. Cough hard, shaking, causing soreness in epigastric region. Expectoration with every cough—thick, lumpy, tasteless. Took *Oxygen*$^{1m}$ at night. Slept well and felt very much better in morning; cough nearly gone. Says it cured more quickly than any remedy he had ever taken, and makes him feel better.

(2). A patient was passing quantities of uric acid like ruby sand. *Oxygen*$^{cm}$ (Swan) removed it in three days.

(3). In HOMŒOPATHIC PHYSICIAN, vol. V, p. 403, Dr. Berridge records a case of cough excited by tickling in throat and causing soreness of chest, occurring between two and three A. M., and better when lying on the back; cured by one dose of *Oxygen*$^{cm}$ (Swan).

(4). At page 44 of Hering's *Analytical Repertory of Symptoms of the Mind* it is stated that *Oxygen* has periodical symptoms every day earlier, and *Hydrogen* every day later. These provings or cures should be published.

